# Counselling in Fetal Medicine: Uncomplicated Twin Pregnancies

**DOI:** 10.3390/jcm13237355

**Published:** 2024-12-03

**Authors:** Filomena Giulia Sileo, Sara Sorrenti, Antonella Giancotti, Daniele Di Mascio, Valentina D’Ambrosio, Fabrizio Zullo, Elena D’Alberti, Martina Derme, Ilenia Mappa, Emma Bertucci, Antonio La Marca, Francesco D’Antonio, Giuseppe Rizzo, Asma Khalil

**Affiliations:** 1 Prenatal Medicine Unit, Department of Medical and Surgical Sciences for Mother, Child and Adult, University of Modena and Reggio Emilia, 41121 Modena, Italyemma.bertucci@unimore.it (E.B.); antonio.lamarca@unimore.it (A.L.M.); 2 Department of Maternal and Child Health and Urological Sciences, Sapienza University of Rome, 00185 Rome, Italy; sara.sorrenti@live.it (S.S.); antonella.giancotti@uniroma1.it (A.G.); dr.valentina.dambrosio@gmail.com (V.D.); fabrizio.zullo@uniroma1.it (F.Z.); elena.dalberti@uniroma1.it (E.D.); martina.derme@uniroma1.it (M.D.); giuseppe.rizzo@uniroma1.it (G.R.); 3 Department of Obstetrics and Gynecology, Università di Roma Tor Vergata, 00133 Rome, Italy; mappa.ile@gmail.com; 4 Center for Fetal Care and High-Risk Pregnancy, Department of Obstetrics and Gynecology, University of Chieti, 66100 Chieti, Italy; dantoniofra@gmail.com; 5 Vascular Biology Research Centre, Molecular and Clinical Sciences Research Institute, St George’s University of London, London SW17 0RE, UK; asmakhalil79@googlemail.com; 6 Fetal Medicine Unit, St George’s Hospital, London SW17 0QT, UK

**Keywords:** twin pregnancy, monochorionic, diamniotic, uncomplicated pregnancy, MCDA, DCDA

## Abstract

Twin pregnancies account for 3% of all pregnancies and they are burdened by higher morbidity and mortality compared to singletons. The role of ultrasound in the screening, diagnosis and management of possible complications of twin pregnancies has been widely investigated in the current literature. However, despite the progress that have been made in the last decades regarding treatment and evidence-based management of complications, twin pregnancies remain at higher risk of adverse outcomes, requiring therefore dedicated surveillance. Thorough counselling regarding the risks and prenatal care should be offered to all future parents of twin pregnancies. This review aims to summarize the current evidence regarding the management of uncomplicated dichorionic and monochorionic pregnancies.

## 1. Introduction

Twin pregnancies are considered as high-risk pregnancies because they bare a higher risk of developing maternal and perinatal adverse outcomes compared to singletons. Twins account for 3% of live births in the United States, with approximately 70% dichorionic and 30% monochorionic pregnancies [1].

The management of such pregnancies varies according to chorionicity and amnionicity; therefore an early diagnosis is essential for providing appropriate care. It is mandatory for maternal–fetal practitioners to properly counsel patients about correct management in the case of uncomplicated twin pregnancies; complicated cases deserve different prenatal care in third-level referral centers.

This review aims to offer a guide to counselling in clinical practice for obstetricians in the case of uncomplicated twin pregnancies.

## 2. How Can We Determine Chorionicity and Amnionicity on Ultrasound?

The management of multiple pregnancies requires a correct diagnosis of chorionicity and amnionicity, in order to identify high-risk pregnancies and to refer monochorionic (MC) twin pregnancies to a tertiary center.

Before 10 weeks, it is possible to distinguish monochorionic pregnancies from dichorionic, based on the number of gestational sacs: a single gestational sac containing two embryos suggests monochorionic twins, while the presence of two distinct gestational sacs suggests a dichorionic pregnancy [2].

However, the most suitable time for this evaluation is at the time of the routine combined screening, from 11 to 14 weeks [3,4,5,6], when the amnion and chorion have not yet fused. For each weekly increase in gestational age, the odds of misclassification rise by 10% [2].

The ultrasound characteristics that should be evaluated to distinguish dichorionic pregnancies from monochorionic pregnancies include the appearance of the membrane attachment to the placenta, the number of placentas, the thickness of the inter-twin membrane [5], and fetal sex, via transabdominal or transvaginal ultrasound.

The most accurate and reproducible method to determine chorionicity is the combined use of T/λ-signs and number of placentas in the first trimester, with a sensitivity and specificity of 100% and 99.8%, respectively [5]. In dichorionic pregnancies, there are two placental masses or, in case of adjacent placental masses, the presence of a triangular tissue projection extending from the base of the inter-twin membrane, which presents a characteristic image of the Greek letter ‘lambda’, or twin peak sign [7]. In monochorionic pregnancies, instead, the inter-twin membrane inserts perpendicularly in the placental plate, presenting an image of the “T-sign” [7].

If the center is uncertain about chorionicity, clinicians should refer the patient for a second opinion from a tertiary referral center and, if it is still not possible to determine chorionicity, the pregnancy should be classified as monochorionic, given the increased risk of complications and pregnancy loss [4,8].

After chorionicity is defined, amnionicity should be determined and documented. The presence of inter-twin amniotic membrane is indicative of diamniotic twins, while its absence indicates monoamniotic twins [7].

## 3. How Can We Label Twins Prenatally?

There are many protocols for labeling twins, such as fetal presentation, sac position, and placental site.

Usually, obstetricians define the position of each twin in relation to the maternal cervix, labeling according to their site, defined as either lateral (left/right) or vertical (upper and lower) orientation [4].

One study proposed a consistent method of twin identification based on the relation between the gestational sac and maternal cervix [9]. In this study, authors stated that the fetus contained in the gestational sac closest to the internal os at the 11–14-week ultrasound assessment should be labelled as twin one. According to the longitudinal axis of the uterus, the twins may have a vertical orientation (right/left), as appears in 90.9% of cases, or a horizontal orientation (inferior/superior) as appears in the remaining 9.1% of pregnancies. The aforementioned technique should be adopted as the preferred method of twin labeling because the position of the gestational sac relative to the cervix remains constant throughout the pregnancy, while the position of either fetus relative to the cervix can change considerably, especially in early pregnancy [9].

The only limitation of this technique is in monochorionic monoamniotic (MCMA) twin pregnancies, where the lack of an inter-twin membrane results in continuously variable fetal position. In these cases, the description of cord insertions in the placenta might be useful for identification of the twins regardless of their changing position in the uterine cavity.

Correct labeling is essential for several clinical reasons: to identify the possible selective growth restriction of each twin, to ensure that invasive prenatal diagnosis or selective fetal reduction is performed on the correct twin and at birth and, in the event of anomalies that cannot be directly inspected, to ensure adequate treatment of the affected twin.

However, it has been highlighted that there is discordance between the prenatal ultrasound order and the birth order of the twins, from about 15.7% (95% CI, 10–25%) [9] to 36% (95% CI, 33.9–38.0) [10], as reported in various studies. Moreover, the greater likelihood of perinatal twin switch is significantly higher for twins delivered by caesarean section (20.3%) compared with those delivered vaginally (5.9%) [9], and this can be partly explained by the fact that Cesarean section allows access to the lower uterine segment and ignores the relationship between the amniotic sac and the maternal cervix, giving preferential access to Twin 2 [10].

Nevertheless, it is important to inform parents of the potential discordance between prenatal and postnatal twin order so that this event is not perceived as an error on the part of their carers [10].

## 4. How Can We Estimate the Risk of Aneuploidies in Twins?

Screening for Trisomy 21 (T21) in twins is complicated by several factors: the zygosity, which cannot be established in dichorionic pregnancies with same gender twins; the biochemistry, including the dosage of pregnancy-associated plasma protein A (PAPP-A) and beta-human chorionic gonadotropin (β-hCG) in maternal blood, which is not fetus-specific and needs to be adjusted for chorionicity; and the lower rate of T21 affected fetuses in twin pregnancies, which do not allow a specific distribution of pathological serum markers [11,12,13]. However, a recent meta-analysis reported that the combined test in twins has a detection rate (DR) of 89.3% (95% CI 79.7–94.7%) with a false positive rate (FPR) of 5.4% (95% CI 4.3–6.7%) with a good performance of the test (summary receiver operating characteristic area under the curve 0.817) [14]. Therefore, it is comparable results achieved in singletons [4,14].

According to the International Society of Ultrasound in Obstetrics and Gynecology (ISUOG) guidelines, the risk for Trisomy 21 (T21) in twin pregnancies can be estimated in the first trimester using the combined test (nuchal translucency (NT) combined with beta-human chorionic gonadotropin level and pregnancy-associated plasma protein-A level); as an alternative, NT and maternal age only can be used, but with an increase in false positive results [4]. In case of spontaneous reduction of the pregnancy to singleton (i.e., the phenomenon of the “vanishing twin”) with a fetal pole still measurable, NT alone with maternal age should be the preferred method in order to reduce the potential residual effect of the second pregnancy on the biomarkers [4,15].

### 4.1. Dichorionic Diamniotic Pregnancy

Dichorionic (DC) twin pregnancies are considered as dizygotic pregnancies in the estimation of T21 risk, since only 10% result from an early splitting of a single embryo [4]. The probability of both fetuses being affected is very low, so the risk for each fetus is independent from the risk for the other. The calculation, therefore, should be based on the sum of the risks of each NT measurement multiplied by the Likelihood Ratio derived from the serum markers [11]. The sensitivity of the test reaches 86.2% (95% CI 72.8–93.6%) and the specificity is 95.2% (95% CI 94.2–96%) [14].

### 4.2. Monochorionic Diamniotic Pregnancy

Monochorionic (MC) twin pregnancies are monozygotic by definition; apart from the rare exception of post-zygotic nondisjunction, MC twins should be considered as sharing the same karyotype. The risk should be estimated per pregnancy rather than per fetus, as both fetuses will be affected or unaffected; therefore, it is appropriate to combine the average of the two NT measurements with the serum markers, and a single risk estimate should be calculated [11]. The sensitivity of the test has been reported as 87.4% (95% CI 52.6–97.7) and the specificity 95.4 (95% CI 94.3–96.3%) [14]. An enlarged NT in MC twin pregnancies can also be a predictor of twin-to-twin transfusion syndrome (TTTS) [16].

## 5. What Is the Role of Non-Invasive Prenatal Testing (NIPT) in Twins?

NIPT can be useful in twin pregnancies because of higher chance of aneuploidy and greater risks of miscarriage in the case of invasive procedures.

A recent meta-analysis by Gil et al., including only prospective studies with follow-up in at least 85% of cases, reported a DR of 98.2% (95% CI, 83.2–99.8%) and a false positive rate (FPR) of 0.05% (95% CI, 0.01–0.26%) for trisomy 21, a DR of 88.9% (95% CI, 64.8–97.2%) and FPR of 0.03% (95% CI, 0.00–0.33%) for trisomy 18, and a DR of 66.7% (2/3) and FPR of 0.19% (5/2569) for trisomy 13 [17] in twin pregnancies.

In twin pregnancies, the interpretation of NIPT results may be challenging; in dizygotic twins, usually only one fetus is likely to be affected, and the percentage of cell-free DNA (cfDNA) in the fetuses can vary by nearly two-fold [18]. The risk is that, if the fetal fraction of the affected twin is below the threshold of 4%, which allows a satisfactory analysis, while the fetal fraction of the normal co-twin is high enough to make the total fraction satisfactory, the aneuploidy might not be identified [19,20]. To minimize the risk of false-negative results, fetal fraction should be estimated per twin; this approach, however, is associated with higher failure rates. In particular, Bevilacqua et al. reported a 5.6% rate of failure in twins [20], while Galeva et al. reported a 11.3% and 4.9% in DC and MC twin pregnancies, respectively [21]. Several factors are associated with failure of NIPT, such as high maternal weight, in vitro fertilization (IVF) conception [20], black or South Asian racial origin, advanced maternal age [21], and also small placental mass, which is associated with trisomy 13, 18 and triploidy [22]. In the case of a failed test, a repeated sample will provide a result in half to two-thirds of cases [21].

A negative or low-risk NIPT is usually a very reassuring result. A positive or high-risk NIPT result should be confirmed by invasive testing, which can be performed with chorionic villous sampling for T21 and for T18 and T13 showing fetal features of the trisomy, or with amniocentesis to avoid erroneous results due to placental-confined mosaicism [17].

In case of repeated failure of the test, which might be caused by low fetal fraction and might reveal an underlying chromosomal abnormality, the decision in favour of or against invasive testing should depend on the risk from prior screening and the results of detailed ultrasound examination looking for specific features of trisomies or triploidy [21].

## 6. What Is the Risk of Miscarriage After Invasive Prenatal Testing in Twin Pregnancies?

Invasive prenatal testing, such as amniocentesis and chorionic villus sampling (CVS), can be offered to patients with twin pregnancies with the same indications as in singletons (maternal age, an abnormal finding in non-invasive prenatal testing). The risk of procedure-related fetal loss is the object of a recent meta-analysis, which showed that the rate of miscarriage in pregnancies undergoing amniocentesis was 2.4% (95% CI, 1.4–3.6%; 83/2713); in particular, in DC pregnancies the rate of fetal loss was 2.3% (95% CI, 0.9–4.1%; 38/1431); similarly, in MC pregnancies the rate of fetal loss was 2.3% (95% CI, 0.1–6.4%; 12/278) [23]. In the case of chorionic villus sampling, the overall miscarriage rate was reported at 2.0% (95% CI, 0.0–6.5%; 8/349); these data were available only in dichorionic pregnancies. Other complications related to invasive procedures include infection and premature preterm rupture of membranes [23,24].

Most operators use a double uterine entry for performing both amniocentesis and CVS in twin pregnancies; the studies included in the aforementioned meta-analysis mostly report experiences of double-needle techniques; only a few studies used a single-needle insertion technique [23]. Current evidence regarding the different risks related to single- or double-needle technique is scarce. Another meta-analysis reported no difference in the rates of miscarriage for the two methods after both CVS (RR 1.58, 95% CI 0.27–9.11) and amniocentesis (RR 0.57, 95% CI 0.06–5.30) [25].

## 7. What Is CRL Discrepancy?

Crown-rump length (CRL) is used in both singleton and twin pregnancies in the first trimester ultrasound scan to assess the correct gestational age. According to current guidelines, in spontaneous twin pregnancies, the gestational age is defined based on the CRL of the larger twin [4].

CRL discordance (%) is calculated as 100 × (larger CRL − smaller CRL)/larger CRL. A meta-analysis showed that CRL discordance between 7 + 0 and 9 + 6 weeks of gestation is predictive of single fetal loss occurring at 11–14 weeks of gestation [26]. Moreover, a significant association between the increase in the degree of embryonic discordance and the likelihood of early fetal loss was found [26].

CRL discordance detected at 11–14 weeks of gestation is associated with an increased risk of adverse pregnancy outcome (fetal and perinatal loss, fetal loss at ≥24 weeks, birth-weight discordance, preterm delivery at <34 weeks and fetal anomalies) [27]. There is no established threshold for CRL discordance in the literature; however, in a meta-analysis by D’Antonio et al., a CRL discordance ≥ 10% between the two fetuses was considered significant, since this usually represents the 90th–95th centile of the population analyzed [27]. The same meta-analysis concluded that the accuracy of CRL discordance in predicting adverse outcome is poor [27], and this was confirmed in both MC and DC twin pregnancies [28]. In MC twin pregnancies, another meta-analysis found an association between intertwin CRL discrepancy ≥ 10%, intertwin NT discrepancy, NT > 95th percentile, or abnormal DV flow on first-trimester ultrasound examination and an increased risk of developing TTTS [29].

## 8. What Is NT Discrepancy?

The aforementioned meta-analysis has demonstrated that MC twin pregnancies with inter-twin NT discrepancy detected on first-trimester ultrasound examination are at significantly increased risk of developing TTTS, with low sensitivity 52.8% (95% CI, 43.8–61.7%) but relatively good specificity 72.5% (95% CI, 61.7–82.0%) [29]. In the literature, the most commonly reported cut-off of inter-twin NT discrepancy is 20%; however, NT discrepancy calculated as percentage of the smaller NT value, or absolute value ≥ 0.6 mm can also be used [29].

Discordance in NT of ≥20% is found in around 25% of MC twin pregnancies and the risk of early fetal death or development of severe TTTS in this group is more than 30% [16]. If the discordance is less than 20%, the risk of complications was found to be less than 10% [16].

In both MC and DC twin pregnancies, the management of a pregnancy with NT discordance ≥20% should be discussed with a fetal medicine expert and following management should include a detailed ultrasound assessment and testing for karyotype abnormalities [4].

## 9. How Often Should Twin Pregnancies Be Assessed on Ultrasound?

**Figure 1:**
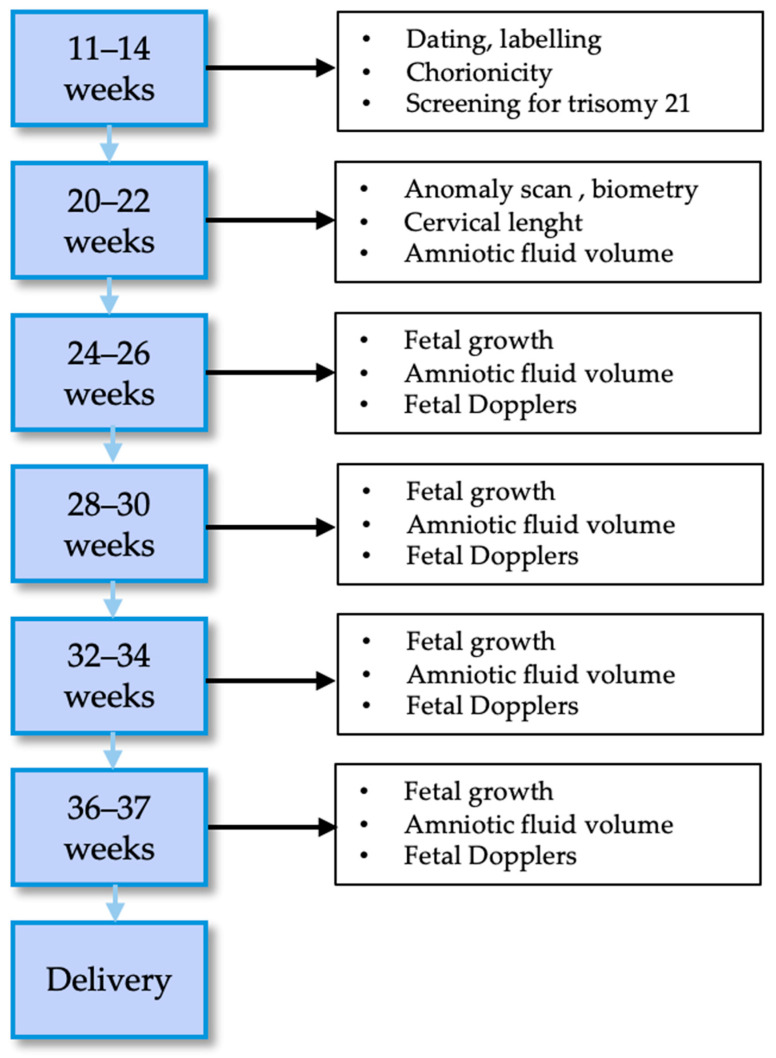
Management of dichorionic diamniotic uncomplicated pregnancies.

**Figure 2:**
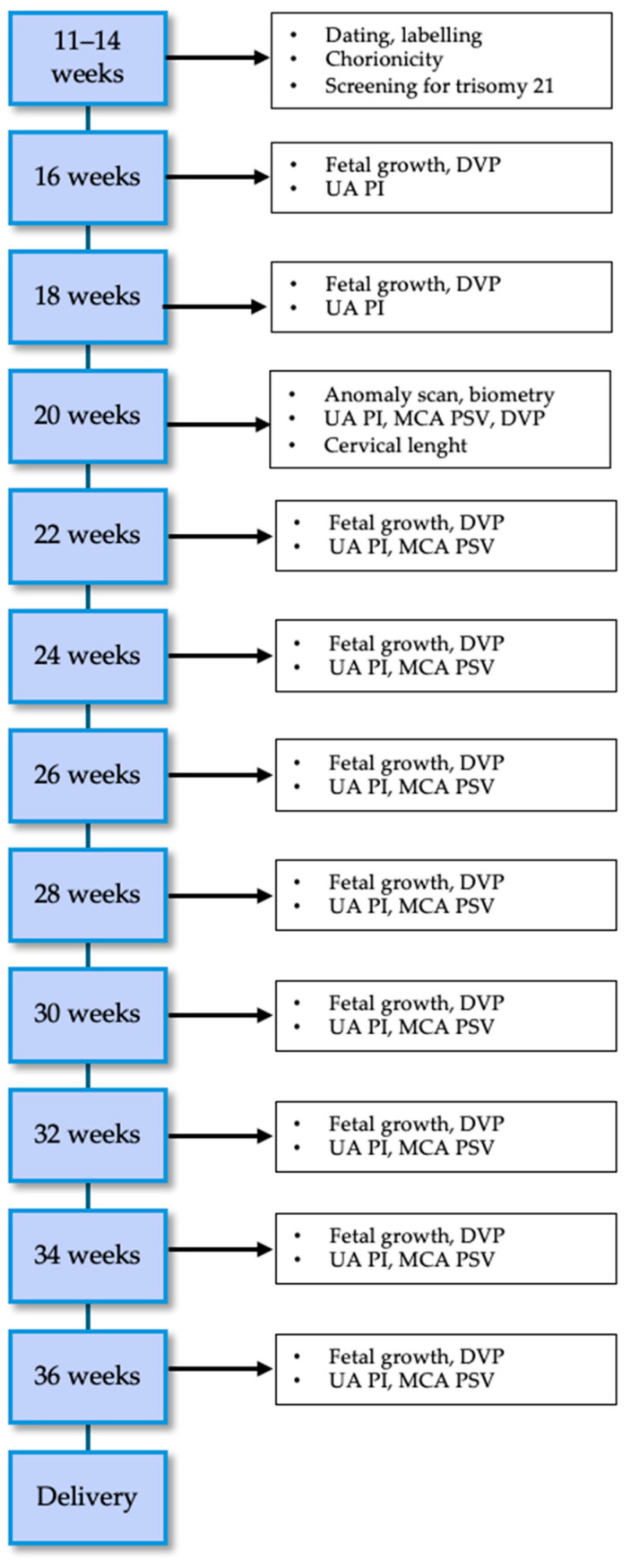
Management of monochorionic diamniotic uncomplicated pregnancies. DVP = deepest vertical pocket; UA = umbilical artery; PI = Pulsatility Index; MCA = Middle Cerebral Artery; PSV = Peak Systolic Velocity.

Every twin pregnancy should be routinely scanned in the first trimester to confirm viability, establish gestational age, assess chorionicity and amnionicity, and offer first-trimester aneuploidy screening [4,30]. The frequency of subsequent ultrasounds and their content will then depend on chorionicity and are shown in Figure 1 for DC and Figure 2 for MC twin pregnancies according to ISUOG guidelines [4].

### 9.1. Dichorionic Diamniotic Pregnancy

Dichorionicity is known to confer lower risk of perinatal complications compared to monochorionicity, with a higher gestational age at delivery and lower rates of congenital anomalies [31,32]. However, DCDA pregnancies may be complicated by fetal growth restriction because of the different genetic potential, noncentral placental cord insertion or differences in uteroplacental efficiency. At present, there is little evidence in the literature on the frequency of ultrasounds in DCDA pregnancies, although several societies recommend examinations every four weeks [4,33]. In the Esprit study, the outcomes of DCDA pregnancies monitored every 2 weeks were excellent and the same authors demonstrated with a secondary analysis that the rate of perinatal complications in DCDA would have possibly been higher with a 4-week interval between ultrasounds because of a reduction in the detection of FGR and abnormal Dopplers compared to 2 week intervals [31]. This is probably a common-sense finding: the increase in the frequency of scans increases the detection of abnormal findings. However, there are also disadvantages of this policy: the need for more resources with a huge impact on health economics, but also implications for healthy co-twins, who could possibly be harmed by an increase in iatrogenic deliveries [34].

### 9.2. Monochorionic Diamniotic Pregnancies

MCDA pregnancies are at higher risk of perinatal complications compared to DCDA pregnancies; moreover, they may develop several complications which are typical of MCDA pregnancies due to the vascular architecture of monochorionic placenta, such as twin-to-twin transfusion syndrome (TTTS), twin–anemia–polycythemia syndrome (TAPS), and selective fetal growth restriction (sFGR) [34,35]. The ISUOG recommendations recommend scanning of these pregnancies every two weeks, since longer intervals have been found to be associated with a more severe presentation of these complications and therefore poorer outcomes [36].

## 10. What Is Weight Discordance?

Birthweight (BW) discordance in twin pregnancies has been associated with worse perinatal outcomes irrespective of chorionicity. It is calculated as 100 × (birth weight of larger twin − birth weight of smaller twin)/birth weight of larger twin [37].

Although a certain degree of growth discordance is considered a normal physiological variation, a higher degree of discordance has been associated with increased perinatal mortality and morbidity [38,39,40,41,42,43,44]. According to a recent meta-analysis, both DC and MC twin pregnancies discordant for fetal growth are at higher risk of IUD (but not of neonatal death, NND) compared with pregnancies with concordant BW, with increasing risk with higher BW discordance [45]. According to the National Institute for Health and Care Excellence (NICE) guidelines, a discordance of at least 20% between twins is considered significant [6]. Moreover, the same meta-analysis showed that the risk of IUD is higher when at least one fetus is small for gestational age (SGA). NND risk was only increased in MC pregnancies with ≥25% BW discordance [45]. BW discordance in twins was also associated with higher neonatal morbidity (including neurological, respiratory, and infectious morbidities, abnormal acid-base status and necrotizing enterocolitis); this association was found to be strong for DC pregnancies but not for MC pregnancies, due to the low power of the analysis because of the small number of included studies [39].

Second-trimester ultrasound discordance (either in estimated fetal weight (EFW) or abdominal circumference (AC)) has not been demonstrated to be predictive of subsequent adverse perinatal outcomes in both MC and DC twins [46]. When patterns of intertwin discordance in AC and EFW across the second and third trimesters were analysed, the expected range of intertwin discordance showed differences not only between DC and MC twin pregnancies, but also with gestational age [43]. In fact, the exact cut-off of discordance for the referral to a tertiary center, and the optimal management and time for delivery have not yet been established [37]. In the third trimester, when discordant growth is detected, antepartum surveillance should be started, although no protocol has been proposed [37]. Optimal delivery time has not been established in these particular cases. In discordant DC pregnancies, when antenatal surveillance is reassuring, evidence is scarce in recommending iatrogenic preterm birth because of the discordance. When delivery is indicated because of non-reassuring antenatal findings for one twin, parents should be informed of the effects of iatrogenic preterm birth on the co-twin and that there is no risk in the case of sudden IUD of one twin [37].

## 11. How Can We Quantify the Risk of Preterm Birth in Twins?

Twin pregnancies are commonly associated with preterm birth (PTB) [47], which is one of the main causes of perinatal adverse outcome in these pregnancies; PTB before 37 weeks occurs in 50% of twin pregnancies and approximately 14% of PTB occurs < 33 weeks [48].

The two most common methods of prediction of PTB in both singleton and twin pregnancies are cervical length (CL) and fetal fibronectin (fFN).

### 11.1. Cervical Length

A single transvaginal measurement of CL at 20–24 weeks of gestation is a good predictor of PTB at <28, <32 and <34 weeks in asymptomatic women with twin pregnancy. In particular, a CL ≤ 25 mm has shown a pooled positive likelihood ratio of 9.6 for the prediction of PTB before 28 weeks [49,50], while the accuracy of predicting PTB < 37 weeks was low.

Among women with a history of preterm labour, the measurement of CL has limited predictive accuracy for preterm birth at <34 and <37 weeks of gestation (pooled sensitivities, specificities, and positive and negative likelihood ratios ranging between 49 and 79%, 32 and 74%, 1.2 and 1.9, and 0.7, respectively) [49,50].

A recent multicenter retrospective study on the performance of transvaginal ultrasound CL according to chorionicity showed that, in monochorionic twins, any given CL was associated with lower gestational age at delivery compared to dichorionic ones (about 2 weeks earlier) [51].

Several studies also evaluated the predictive value for PTB of CL change over time, but this was not superior to a single measurement at 18–24 weeks [49,52,53,54].

Additionally, the presence of cervical funneling during evaluation of CL has no predictive value for PTB in both symptomatic and asymptomatic women, as evaluated by Conde-Aguelo et al. in their meta-analysis [50]. Symptoms might include uterine contractile activity and cervical dilatation.

### 11.2. Cervicovaginal Fetal Fibronectin

The accuracy cervicovaginal fetal fibronectin (fFN) in predicting PTB < 32, <34 and <37 weeks was evaluated in a systematic review and meta-analysis by Conde-Aguelo et al.; the test showed limited value in asymptomatic women [55]. Among symptomatic women, fFN was most accurate in predicting spontaneous PTB within 7 days from the test [50,55].

### 11.3. Combination of CL and fFN

Some studies evaluated the accuracy of CL and fFN in asymptomatic [56,57] and symptomatic women [58,59]; there is no evidence that the combination or the sequential use of the two tests is a better predictor than each test alone in both asymptomatic and symptomatic women [49].

## 12. What Are the Risks of a Single Intra-Uterine Death in Twins?

Single intra-uterine death (sIUD) occurs in approximately 6% of twin pregnancies [60]. In the first trimester, this phenomenon is known as “vanishing twin”, occurs in approximately 36% of twins, and is rarely associated with adverse outcomes [4]. On the other hand, sIUD after 14 weeks of gestation carries a higher risk of adverse events for the surviving twin. Chorionicity is determinant for the rate of fetal loss, outcomes for the co-twin and management of the pregnancy in these circumstances [35]). The shared placenta in MCDA pregnancies, in fact, exposes the co-twin to increased risk of death and morbidity.

In particular, possible complications associated with sIUD include death of the co-twin, preterm birth (both spontaneous and iatrogenic) and long-term sequelae, most commonly brain injury (with antenatal and/or postnatal diagnosis) [4]. A recent meta-analysis by Mackie et al. evaluated the prognosis of the surviving co-twin following spontaneous sIUD according to chorionicity [61]. The rate of intrauterine death (OR 2.06 (95% CI 1.14–3.71) *p* = 0.016) and brain injury (5.41 (95% CI 1.03, 28.58) *p* = 0.047) of the surviving twin was higher in MC pregnancies than in DC [61]. This was particularly evident in the case of sIUD before 28 weeks, independently from TTTS or sFGR [61]. On the other hand, no difference was found between MCDA and DCDA pregnancies in the rate of PTB, neurodevelopmental comorbidity and neonatal death of the surviving twin [61]. The rate of abnormal antenatal brain imaging in MCDA pregnancy has been reported to reach 20% [61]; typical MRI findings after sIUD are hypoxic–ischemic parenchymal lesions (intraventricular hemorrhage) and malformations of cortical development, such as polymicrogyria [62,63]. These lesions are thought to be caused by acute cerebral hypoperfusion because of exsanguination of the survivor into the dead fetus, or thromboembolic events [64].

Given this substantial risk of adverse outcomes for the co-twin, management of these pregnancies is challenging, and evidence is scarce and mainly deriving from small series with different protocols [65].

In DCDA pregnancies, conservative management with close surveillance and delivery close to term should be the preferred option, considering steroid prophylaxis in case of sIUD after viability due to the risk of spontaneous PTB [65,66].

Management of MCDA pregnancies with sIUD is particularly challenging; steroid prophylaxis is indicated after viability due to the risk of spontaneous PTB but also for iatrogenic early delivery due to fetal compromise of the surviving twin. Optimal gestational age for delivery has not been established; the majority of these pregnancies are delivered earlier because of clinicians’ concern, although this policy not only does not reduce the associated neonatal morbidity but adds risks of prematurity [65].

## 13. When Should Uncomplicated Twin Pregnancies Be Delivered?

Twin pregnancies have a fourfold higher risk of stillbirth compared with singleton [67,68]; when distinguishing twin pregnancy according to chorionicity, there is a thirteen and fivefold increase in stillbirth rates in monochorionic and dichorionic twin pregnancies, respectively [69]. According to Wood et al., the risk of stillbirth in MCDA pregnancy is bimodal, with most stillbirths occurring before 29 weeks of gestation and then with the risk increasing again after 36 weeks of gestation [70].

In order to prevent late stillbirths, there has been an increase in preterm delivery of uncomplicated twin pregnancies, although this policy is associated with an increase in neonatal complications associated with prematurity [71].

Guidelines from the American College of Obstetricians and Gynecologists (ACOG) recommend delivery from 38 + 0 to 38 + 6 in uncomplicated dichorionic pregnancies and from 34 + 0 to 37 + 6 in uncomplicated monochorionic diamniotic pregnancies [72], whereas the National Institute for Health and Care Excellence (NICE) guidelines recommend delivery at 37 weeks for dichorionic and at 36 weeks for monochorionic pregnancies [33].

## 14. What Is the Optimal Route of Delivery in Twin Pregnancies?

The optimal route of delivery in women with twin gestations depends on the type of twins, fetal presentations, gestational age and experience of the clinician performing the delivery. Current guidelines [33,72] agree that, regardless of the presentation of the second twin, vaginal delivery is a reasonable option and should be considered in uncomplicated DC and MC diamniotic twin pregnancies after 32 weeks, as long as there are no obstetric contraindications to labour, the first baby is in a cephalic presentation and there is no significant size discordance between the twins. Nevertheless, more than a third of women who plan a vaginal birth end up having a caesarean section (CS).

These recommendations are based on the evidence deriving from the Twin Birth Study [73] and supported by the Cochrane review published in 2015 [74]. This large randomized clinical trial suggested that planned CS did not reduce the risk of fetal or neonatal death or serious neonatal morbidity, compared with planned vaginal delivery (2.2% and 1.9%, respectively, OR 1.16; 95% CI, 0.77–1.74; *p* = 0.49) in pregnancies with cephalic presenting twins between 32 and 38 weeks of gestation [73].

Recently, a secondary analysis of the Twin Birth Study [75] compared neonatal outcomes of women who presented in spontaneous labor in the two arms, suggesting that there is no statistically significant difference in neonatal composite outcomes between women who presented in spontaneous labor planning to deliver vaginally or by CS. In the literature, evidence suggests that the spontaneous twin birth of the second non-cephalic fetus has a greater success rate if performed by breech extraction rather than external cephalic version (95% vs. 42%) [76,77]; therefore, it can be concluded that breech extraction is the optimal procedure for the non-cephalic second twin.

It is indicated to offer CS to women if the first twin is not cephalic at the time of planned birth [35]. These recommendations are based on the evidence of The Term Breech Trail [78]. This randomized controlled trial on singleton term breech delivery reported that planned CS reduced the risk of perinatal death or serious neonatal morbidity three-fold (from 5.0% to 1.6%; *p* < 0.001), compared to planned vaginal delivery, without a significant increase in maternal complications. In twin pregnancies, there is a risk of combined delivery (vaginal delivery of the first twin followed by cesarean delivery of the second twin). Peaceman et al. analyzed multiple matched birth data of 450,000 infants and showed that the combined delivery for vertex/vertex and vertex/non vertex twins was performed in 4.2% and 22.6% of cases, respectively [79].

The optimal time interval between delivery of the first and the second twin is unclear. It was previously believed that this interval should be no longer than 30 min, as a prolonged interval placed the second twin at risk of asphyxia from decreased placental circulation [80]. Nowadays, it is believed that the delayed delivery of the second twin in multiple pregnancies can be successful in selected cases [81]. Before 28 weeks of gestation, delayed delivery of the second twin can be an option to prolong the pregnancy until a gestational age, which confers a better prognosis and a better perinatal outcome for the second twin, while informing the patient about the risks and benefits.

During labour, in women with a twin pregnancy and at more than 26 weeks, continuous and simultaneous cardiotocography is recommended [33]. It is possible to offer an epidural to women with a twin pregnancy who choose to have a vaginal birth [33]. Multiple pregnancy is a risk factor for uterine atony, postpartum hemorrhage, and emergent hysterectomy at delivery, therefore an active management of the third stage of labour should be offered [33].

## 15. Conclusions

It is of great importance to correctly define chorionicity and amnionicity in the first trimester in order to properly counsel patients regarding the risks and the type of surveillance required for dichorionic and monochorionic pregnancies. In addition, labelling of the twins using the respective position of the gestational sac in relation to the maternal cervix may reduce the risk of errors in the monitoring of growth and Dopplers throughout pregnancy. Dichorionic pregnancies need 4-weekly monitoring, whereas monochorionic pregnancies need 2-weekly assessment because of the higher risk of complications.

The screening for aneuploidies may be performed using the combination of nuchal translucency and biomarkers (PAPP-A and β-hCG) or using the NIPT, which has demonstrated high accuracy in twin pregnancies.

Timing of delivery should not exceed 37 weeks in dichorionic and 36 weeks of gestation in monochorionic pregnancies according to the NICE guidelines, whereas ACOG guidelines suggest delivering uncomplicated dichorionic pregnancies not later than 38 + 6 weeks and uncomplicated monochorionic pregnancies not later than 37 + 6 weeks. Vaginal delivery is considered a reasonable option in uncomplicated twin pregnancies after 32 weeks, in the absence of obstetric contraindications to labor, with the presenting fetus in cephalic lie.
